# *Borrelia*, *Rickettsia*, and *Ehrlichia* Species in Bat Ticks, France, 2010

**DOI:** 10.3201/eid1812.111237

**Published:** 2012-12

**Authors:** Cristina Socolovschi, Tahar Kernif, Didier Raoult, Philippe Parola

**Affiliations:** Author affiliations: Unité de Recherche en Maladies Infectieuses et Tropicales Emergentes, Marseille, France (C. Socolovschi, T. Kernif, D. Raoult);; World Health Organization Collaborative Center for Rickettsial Diseases and Other Arthropod-Borne Bacterial Diseases, Marseille (P. Parola)

**Keywords:** Argas vespertilionis, argasid tick, bat tick, bat, ticks, Borrelia, Rickettsia, Ehrlichia, Coxiella burnetii, France, bacteria, vector, reservoir, tickborne, parasites

## Abstract

*Argas vespertilionis*, an argasid tick associated with bats and bat habitats in Europe, Africa, and Asia has been reported to bite humans; however, studies investigating the presence of vector-borne pathogens in these ticks are lacking. Using molecular tools, we tested 5 *A. vespertilionis* ticks collected in 2010 from the floor of a bat-infested attic in southwestern France that had been converted into bedrooms. *Rickettsia* sp. AvBat, a new genotype of spotted fever group rickettsiae, was detected and cultivated from 3 of the 5 ticks. A new species of the *Ehrlichia canis* group, *Ehrlichia* sp. AvBat, was also detected in 3 ticks. Four ticks were infected with *Borrelia* sp. CPB1, a relapsing fever agent of the *Borrelia* group that caused fatal borreliosis in a bat in the United Kingdom. Further studies are needed to characterize these new agents and determine if the *A. vespertilionis* tick is a vector and/or reservoir of these agents.

## Abstract 2

Physicians should consider infections with these bacteria in patients who may have been bitten by bat ticks.

Ticks are obligate hematophagous arthropods that are considered to be second only to mosquitoes as vectors of agents that cause diseases in humans (1). Ticks parasitize every class of vertebrates in most regions of the world and occasionally bite humans (1). *Argas vespertilionis* (also known as *Carios vespertilionis*) ticks parasitize several bat species around the world, except in the Americas (2,3). Bats are adequate hosts for blood ingestion by ticks because they lack dense fur and have a supply of large blood vessels just below the dermis. In addition, a larval *A. vespertilionis* tick was collected from a dog in Sweden (3). Common habitats for these ticks include cracks and crevices in the walls of bat-infested caves and buildings, tree holes, and any niche occupied by the host. Tick nymphs and adults can bite persons in caves (4,5). *A. vespertilionis* tick populations in Europe and South Africa exist in temperate climates with pronounced seasonal changes and moderate to heavy rainfall.

The role of *A. vespertilionis* ticks as vectors or reservoirs of bacterial, viral, or protozoal pathogens is poorly understood; however, several pathogens have been detected in these ticks. In 1966, *Coxiella burnetii*, the agent of Q fever, was detected in *A. vespertilionis* ticks collected from southern Kazakhstan (6), and in 1973, an arbovirus named Issyk-Kul virus was isolated from bats and *A. vespertilionis* ticks in Kyrgyzstan (7). A few years later, Issyk-Kul virus was isolated from a scientist who had become infected while conducting field work in the Kumsangir district of southern Tajikistan (8). *Candidatus* Babesia vesperuginis showed potential pathogenicity to a bat in the United Kingdom, and the study authors hypothesized that the *A. vespertilionis* tick could be a vector for these protozoa (9). *Borrelia burgdorferi* sensu lato, the infectious agent of Lyme disease, was detected in *A. vespertilionis* ticks that were collected during 1896–1994 and housed at the Natural History Museum in London. Thus, 13/13 ticks collected from bats (mostly pipistrelles) and 12 (75%) of 16 ticks collected from human dwellings had results positive for *B. burgdorferi* s.l. when tested by nested PCR targeting the *ospA* gene (10). However, PCR contaminations were not excluded for these results. It has been stated that this tick is a vector of spirochetes in bats, but no conclusive evidence has supported this hypothesis (2).

The WHO (World Health Organization) Collaborative Center for Rickettsial Diseases and other Arthropod-Borne Bacterial Diseases receives human samples and arthropod specimens from all parts of the world for tick-borne disease diagnosis. The aim of this study was to analyze *A. vespertilionis* ticks for the presence of *Borrelia*, *Rickettsia*, *Bartonella*, and *Ehrlichia* spp. and for *C. burnetii* by using molecular and culture tools.

## Materials and Methods

### Tick Collection

**Figure 1:**
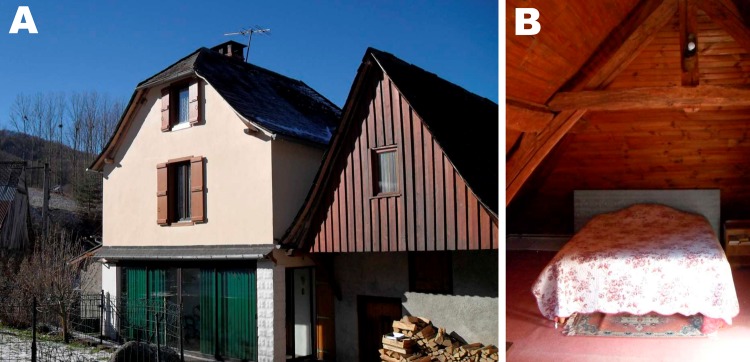
Bat-infested home in Astien, southwestern France, in the Ariège region of the Pyrenees Mountains. *Argas vespertilionis* ticks were collected from the floor of the attic, which had been converted into bedrooms (right).

On July 12, 2010, the owners of a bat-infested home in Astien, France (42°56′18.25′′N, 1°03′54.57′′E; elevation 542 m) found 6 live ticks on the floor of their attic, which had been converted into bedrooms (Figure 1). Astien is located in southwestern France in the Ariège region of the Pyrenees Mountains. The ticks were sent to our laboratory at the WHO Collaborative Center for Rickettsial Diseases and Other Arthropod-Borne Bacterial Diseases (Marseille, France), where we used standard taxonomic keys to identify them morphologically as adult *A. vespertilionis* (Latreille, 1802) ticks (Video) (2). 

**Video:**
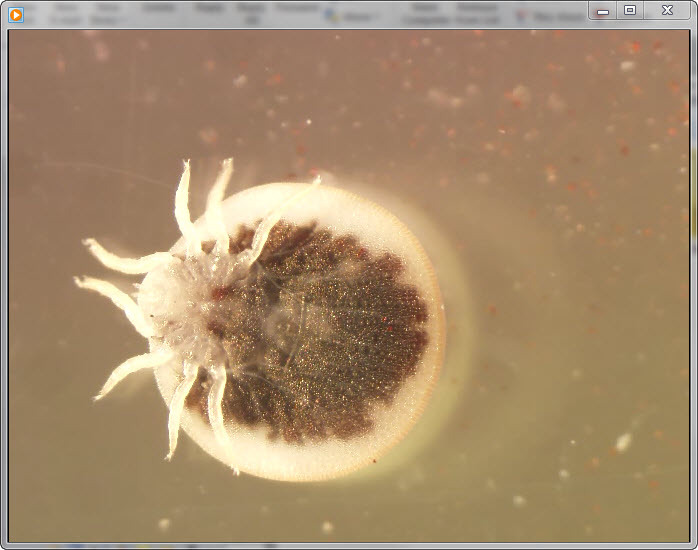
Ventral view of a live adult argasid tick under 10× magnification. This tick species (*Argas vespertilionis*) is associated with bats and bat habitats in Europe, Africa, and Asia. The body outline is circular. The integument is smooth and marked by a fine network of small, irregular cells among which regular, subparallel rows of larger disks radiate. Legs arise from the anterior half of the body and are shorter than the body; coxae are contiguous; and tarsi are tapered and lack dorsal humps (2). The video is 26 seconds long and has no audio.

Direct Video Link: http://streaming.cdc.gov/vod.php?id=7b6a1467dfc5341ead6bbc9ac215fca520121004162012606 Persicargas

### DNA Extraction 

Five of the 6 ticks were washed in a 10% water solution of commercial disinfectant–detergent (Amphomousse; Hydenet S.A., Sainghin-en-Melantois, France), rinsed in sterile water, and placed in a 1% solution of sodium hypochlorite for 10 minutes. The ticks were then rinsed with distilled water and incubated in 70% ethanol for 15 minutes, after which they were rinsed in sterile phosphate-buffered saline, dried on sterile filter paper in a laminar flow hood, and individually crushed in sterile tubes (Eppendorf; Hamburg, Germany). DNA was extracted from one half of each of the 5 ticks by using the QIAamp Tissue Kit (QIAGEN, Hilden, Germany) according to the manufacturer’s instructions. The genomic DNA was stored at 4°C until used as a template in PCR assays. The remaining portion of each tick was kept at –80°C for further analysis. DNA extraction and the molecular identification of all ticks were efficiently achieved by using one molecular system: PCR amplification with sequencing of the 338-bp region of the 12S RNA gene, as described (11). The sixth tick was kept in the tick collection of the WHO Collaborative Center for Rickettsial Diseases and other Arthropod-Borne Bacterial Diseases, Marseille, France.

### Detection of *Rickettsia* spp.

We used quantitative real-time PCR (qPCR) with the 1029 system based on the RC0338 hypothetical protein gene to screen DNA samples from the 5 ticks for all spotted fever group (SFG) rickettsiae (12) (Table). Reactions were performed by using LightCycler 2.0 equipment and software (Roche Diagnostics GmbH, Mannheim, Germany). Master mixes were prepared according to the manufacturer’s instructions. We confirmed rickettsiae-positive results by using conventional PCR with the GeneAmp PCR System 2400 thermal cycler (PerkinElmer, Waltham, MA, USA). We used primers CS2d–CS877f and Rp CS.409p–Rp CS.1258n to amplify and sequence the full-length citrate synthase gene (*gltA)* found in all rickettsiae (20), and we used primers Rr. 190.70 and Rr. 190.701 to amplify and sequence a fragment of the outer membrane protein A (*ompA*) gene (629–632 bp), which encodes a 190-kDa protein (17). We used 2 negative controls for all PCR reactions: 1) PCR mix alone and 2) PCR mix with noninfected *Rhipicephalus sanguineus* tick DNA (free of *Rickettsia*, *Ehrlichia*, *Anaplasma*, *Bartonella*, and *Borrelia* spp. and *C. burnetii*). We used DNA extracted from *R. montanensis* as a positive control for detection of rickettsiae. Amplification products were analyzed after electrophoresis on a 1% agarose gel stained with ethidium bromide.

**Table:** Primers and probes used to detect and confirm the presence of bacteria in *Argas vespertilionis* ticks collected in 2010 from an attic in France*

Bacterial species	Gene target (ref)	Primers and probe
		Screening
*Rickettsia *	RC0338 (12)	1029-F1: 5′-GAM AAA TGA ATT ATA TAC GCC GCA AA-3′ 1029-R1: 5′-ATT ATT KCC AAA TAT TCG TCC TGT AC-3′ 1029–1P: 6FAM-CTC AAG ATA AGT ATG AGT TAA ATG TAA A-TAMRA
			*Borrelia *	16S rRNA (13)	Bor_16S_3_F: 5′ AGC CTT TAA AGC TTC GCT TGT AG 3′ Bor_16S_3_R: 5′ GCC TCC CGT AGG AGT CTG G 3′ Bor_16S_3_P: 6FAM- CCG GCC TGA GAG GGT GAA CGG TAMRA
*Coxiella burnetii*	IS*1111* (14)	IS 1111F: 5′-CAA GAA ACG TAT CGC TGT GGC-3′ IS 1111R: 5′-CAC AGA GCC ACC GTA TGA ATC-3′ IS1111P: 6-FAM-CCG AGT TCG AAA CAA TGA GGG CTG-TAMRA			
*Bartonella *	ITS (15)	Barto ITS3 F: 5′-GAT GCC GGG GAA GGT TTT C-3′ Barto ITS3 R: 5′-GCC TGG GAG GAC TTG AAC CT-3′ Barto ITS3 P: 6 FAM-GCG CGC GCT TGA TAA GCG TG-TAMRA
*Ehrlichia *	16S rRNA (16)	**Erli_16S_F: 5′-GGT-ACC-YAC-AGA-AGA-AGT-CC-3′** **Erli_16S_R: 5′-TAG-CAC-TCA-TCG-TTT-ACA-GC-3′**
		Confirmation
*Rickettsia *	*gltA* (17)	**409D: 5′-CCT ATG GCT ATT ATG CTT GC-3′** **1258R: 5′-ATT GCA AAA AGT ACA GTG AAC A-3′**
*Rickettsia *	*ompA* (17)	**190–70: 5′-ATG GCG AAT ATT TCT CCA AAA-3′** 190–701: 5′- GTT CCG TTAATGGCAGCA TCT-3′ **190–180: 5′- GCA GCG ATA ATG CTG AGT A-3′**
*Borrelia *	16S rRNA (13)	**BF1: 5′-GCT GGC AGT GCG TCT TAA GC-3′** **BR1: 5′-GCT TCG GGT ATC CTC AAC TC-3′**
*Borrelia *	Flagellin (18)	Bor1: 5′- TAA TAC GTC AGC CAT AAA TGC-3′ Bor2: 5′- GCT CTT TGA TCA GTT ATC ATT C-3′
*C. burnetii*	IS*30* (14)	IS30a F: 5′-CGC TGA CCT ACA GAA ATA TGT CC-3′ IS30a R: 5′-GGG GTA AGT AAA TAA TAC CTT CTG G-3′ IS30a P: 6-FAM-CAT GAA GCG ATT TAT CAA TAC GTG TAT GC-TAMRA
*Bartonella*	ITS (15)	**Urbarto1: 5′-CTT-CGT-TTC-TCT-TTC-TTC-A-3′** **Urbarto2: 5′-CTT-CTC-TTC-ACA-ATT-TCA-AT-3′**
*Ehrlichia*	*gltA* (19)	**HER-CS133F: 5′-GGW TTY ATG TCY ACT GCT GC-3′** **HER-CS778R: 5′-GCN CCM CCA TGM GCT GG-3′**

*Ref, reference. Primers in **boldface** were used for sequencing.

The second half of each tick was placed in sterile 1.5-mL plastic tubes, where they were triturated with a sterile micropestle in 600 μL of Rinaldini solution (6.8 g NaCl, 0.4 g KCl, 0.156 g NaH_2_PO_4_, 2.2 g NaHCO_3_, 1.0 g glucose, and 1.0 mg phenol red in 1,000 mL sterile double-distilled water). To isolate *Rickettsia* spp. from the tick solution, we used a shell vial cell culture assay, as described (21). In brief, we inoculated 300 µL of the rickettsia PCR–positive ticks into 7 shell vials containing 1-cm^2^ coverslips on which cell culture lines had been grown. Of the 7 shell vials, 3 contained a coverslip with a monolayer of mouse fibroblasts (L929 cells); 3 contained coverslips with a monolayer of human embryonic lung fibroblasts; and 1 contained a coverslip with cell line XTC-2, derived from *Xenopus laevis*. We did not include antimicrobial drugs in the medium. After the vials were incubated for 8, 15, and 21 days, we performed Gimenez staining and indirect immunofluorescence assays to detect rickettsial organisms in cell culture as described (21). Cultures were considered rickettsiae-positive if staining and assay results were positive. We sampled culture supernatants to identify isolates by standard PCR as described (17,20).

### Detection of *Borrelia* spp.

We used qPCR targeting the 16S rRNA gene, as described (13), to screen DNA samples from the 5 ticks for all *Borrelia* spp. (Table). Samples with borreliae-positive results were confirmed positive by conventional PCR with primers Bf1-Br1 and Bor1-Bor2, which enabled amplification of the 16S rRNA gene fragment and *flaB* gene, respectively (18,22). We sequenced the amplified product as described above. Positive control reactions for each assay incorporated DNA extracted from *Borrelia crocidurae*. We injected the solution of *Borrelia* spp. PCR–positive ticks into 2 tubes with 10 mL of BSKH medium (Sigma-Aldrich, Taufkirchen, Germany) (23) and a 100-µL solution of antimicrobial drugs (product no. A1956 [2 mg phosphomycin (fosfomycin), 5 mg rifampin, and 250 μg amphotericin B per mL in 20% DMSO]; Sigma-Aldrich). Samples were cultivated at 33°C, and once a week we used dark-field microscopy to examine them for the presence of spirochetes. We considered samples to be negative for borreliae if no growth was detected after 8 weeks of incubation.

### Detection of *Bartonella* spp.

We used qPCR to screen DNA samples for a fragment of the *Bartonella* spp. intergenic spacer region between the 16S and 23S rRNA genes (Table). Conventional PCR that amplifies a fragment of the 732-bp intergenic spacer region of *Bartonella* spp. was used to confirm bartonellae-positive results (15). DNA extracted from *B. elizabethae* served as a positive control for detection of bartonellae.

### Detection of *C. burnetii*

We initially detected bacterial DNA by qPCR with *C. burnetii*–specific primers and a probe designed to amplify the IS*1111* gene (14). We used qPCR with primers and a probe designed for the amplification of IS30A spacers to confirm *C. burnetii*–positive results (14). Amplification of both spacers indicated a positive result. In each test, DNA extracted from *C. burnetii* served as a positive control.

### Detection of *Ehrlichia/Anaplasma spp.*

We detected *Ehrlichia*/*Anaplasma* spp. DNA by conventional PCR using primer set EHR16SR– EHR16SD, which amplify a 345-bp fragment of the 16S rRNA gene of ehrlichiae (16). A second PCR that amplified a fragment of the citrate synthase gene of *Ehrlichia* spp. was used, as described (19), to confirm positive amplification results (Table). The amplified products for both genes were sequenced as described above. Two negative controls and 1 positive control (DNA from *A. phagocytophilum)* were included in each test.

### Sequence Analysis

All obtained sequences were assembled and edited by using Auto Assembler software version 1.4 (PerkinElmer). We analyzed sequences by using BLAST (www.ncbi.nlm.nih.gov/blast/Blast.cgi) and compared them with sequences in the GenBank database. We performed multiple sequence alignments by using the ClustalX program (www.clustal.org/clustal2/). Phylogenetic trees were constructed by using the test minimum-evolution tree algorithm in the MEGA5 program (http://megasoftware.net/). Support for the tree nodes was calculated with 100 bootstrap replicates.

## Results

### Tick Identification

The 12S RNA gene from 4 of the 5 *A. vespertilionis* ticks in our study showed 81.4% (253/311 bp) sequence similarity with the *Carios capensis* tick (GenBank accession no. AB075953) and 77.7% (303/390 bp) sequence similarity with the *Ixodes granulatus* tick (GenBank accession no. DQ003012). We submitted the 12S RNA sequence to GenBank (accession no. JX233821); no other 12S sequence for *A. vespertilionis* ticks was available in GenBank.

### Detection of *Rickettsiae* spp.

Of the 5 ticks we tested, 3 (sample nos. 62494, 62497, and 62498) were positive for genus-specific rickettsiae DNA by qPCR. Subsequent sequencing of *gltA* gene amplicons from all positive PCR samples showed that the closest sequences available in GenBank were those for *R. peacockii* (accession no. CP001227), *R. africae* (accession no. CP001612), and *R. conorii* (accession no. AE006914), which showed 99.6% (1,257/1,262 bp), 99.52% (1,256/1,262 bp), and 99.52% (1,256/1,262 bp) sequence identity, respectively. The closest sequences available in GenBank for the *ompA* gene fragment were those for *R. africae* ESF-5 (accession no. CP001612), *Rickettsia* sp. strain S (accession no. RSU43805), and *R. mongolitimonae* BJ-90 strain (accession no. AF179365), which showed 99.34% (611/615 bp), 99.5% (587/590 bp), and 98.67% (594/602 bp) sequence identity, respectively. The sequences for all 3 ticks were identical.

**Figure 2:**
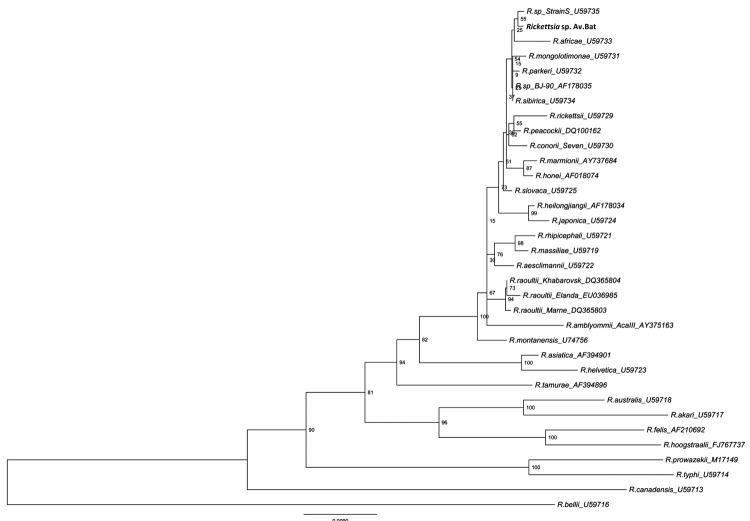
Phylogenetic tree drawn using the minimum evolution method from an alignment of the 1,225-bp *gltA* gene of *Rickettsia* sp. AvBat. Bootstrap values are indicated at the nodes. Scale bar indicates the degree of divergence represented by a given length of branch. **Boldface** indicates the taxonomic position of a new *Rickettsia* sp.

**Figure 3:**
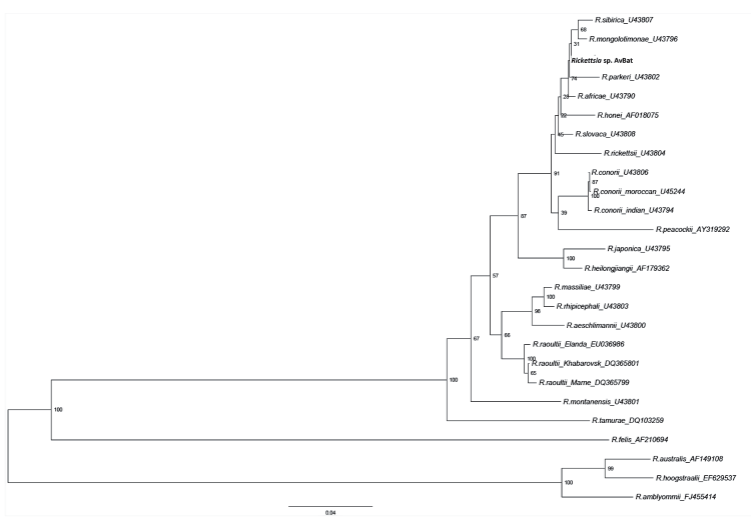
Phylogenetic tree drawn, using the minimum evolution method, from an alignment of the 611-bp *ompA* gene of *Rickettsia* sp. AvBat. Bootstrap values are indicated at the nodes. Scale bar indicates the degree of divergence represented by a given length of branch. **Boldface** indicates the taxonomic position of a new *Rickettsia* sp.

The positive and negative controls produced the expected results. The nucleotide sequence of the full-length *gltA* gene of *Rickettsia* sp. was deposited in GenBank (accession no. JN038177) and named *Rickettsia* sp. AvBat. Maximum parsimony and neighbor-joining analysis of the 1,225-bp *gltA* gene and the presence of the *ompA* gene suggest that *Rickettsia sp.* AvBat be classified as an SFG rickettsiae, with the closest relationships to *Rickettsia* sp*.* strain S (U59735) and *R. africae* (U59733) (Figures 2, 3) (24). On the basis of the guidelines for the classification of new *Rickettsia* spp. (24), the bacterial species we identified shared <99.9% similarity with the full-length *gltA* gene and >98.8% similarity with the partial *ompA* gene.

**Figure 4:**
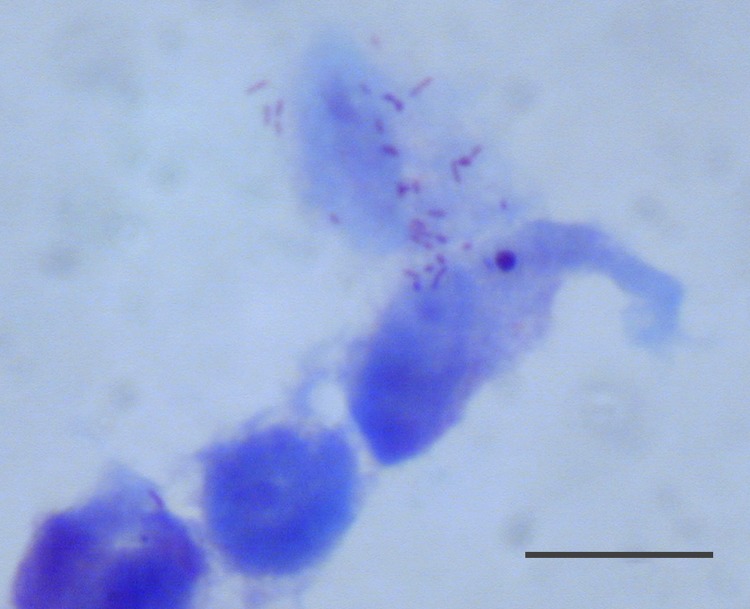
*Rickettsia* sp. AvBat in XTC-2 cell culture with Gimenez staining. Scale bar = 20 μm.

On day 15 of incubation, the shell vial cultures for 2 PCR rickettsiae-positive ticks had Gimenez staining and immunofluorescence assay results positive for *Rickettsia* spp. (Figure 4). The 2 isolates were established in the L929 and XTC-2 cells, respectively (3 passages/cell line).

### Detection of *Borrelia* spp.

**Figure 5:**
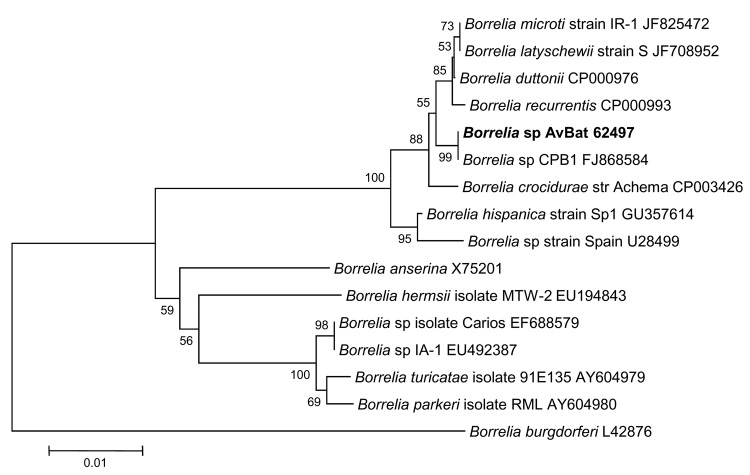
Phylogenetic trees drawn from an alignment of the 736-bp *flaB* gene specific to *Borrelia* spp. by using the minimum evolution method. Bootstrap values are indicated at the nodes. Scale bar indicates the degree of divergence represented by a given length of branch. **Boldface** indicates the position of *Borrelia* sp. AvBat in the phylogenetic tree.

**Figure 6:**
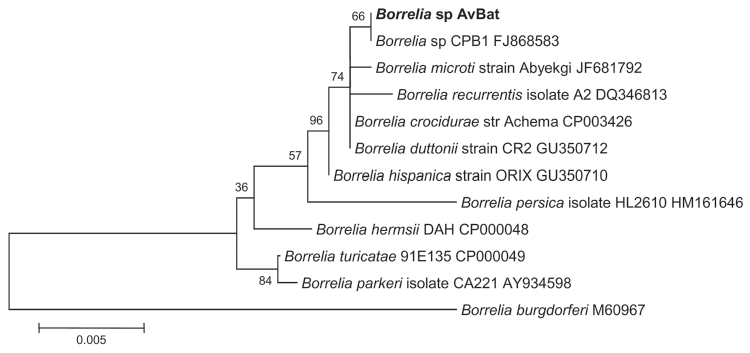
Phylogenetic tree drawn from an alignment of the 1206-bp 16S rRNA gene specific to *Borrelia* spp. by using the minimum evolution method. Bootstrap values are indicated at the nodes. Scale bar indicate the degree of divergence represented by a given length of branch. **Boldface** indicates the position of *Borrelia* sp. AvBat in the phylogenetic tree.

Of the 5 *A. vespertilionis* ticks tested for the presence of *Borrelia* sp. by qPCR, 4 were positive: tick numbers 62494, 62495, 62497, and 62498. We used standard PCR to amplify the *Borrelia* 16S rRNA gene fragment from all 4 positive ticks. For all samples, DNA sequence analyses of the PCR products showed 100% (1,206/1,206 bp) similarity with the 16S rRNA sequence of *Borrelia* sp. CPB1 (GenBank accession no. FJ868583) and 100% (736/736 bp) similarity with the flagellin gene sequence of *Borrelia* sp. CPB1 (GenBank accession no. FJ868584) (25). The bacterial cultures of the borrelia PCR–positive samples did not grow borreliae. Phylogenetic analysis of 2 genes (Figures 5, 6) showed that this *Borrelia* sp. is close to, but distinct from, a cluster containing *B. recurrentis*, *B.* *duttonii*, *B. microtii*, *B. latyschewii*, and *B. crocidurae*.

### Detection of *Ehrlichia/Anaplasma* spp.

Five ticks were tested by standard PCR for the 16S rRNA and *gltA* genes specific for *Ehrlichia*/*Anaplasma*; results were positive for 3 ticks (nos. 62495, 62496, and 62497). Sequence analyses showed 99.4% (344/346 bp) similarity with the 16S rRNA gene of uncultured *Ehrlichia* sp. clone Khabarovsk 1931 (GenBank accession no. FJ966354) and 98.3% similarity with *E. muris* isolate Kh-1550 (GenBank accession no. GU358692). For these 3 ticks, the closest matches to a *gltA* gene fragment in GenBank were with those of *Ehrlichia* sp. HF (accession no. DQ647319), *Ehrlichia* sp. Yamaguchi (accession no. AF304145), and *E. muris* (accession no. AF304144), which had 88.93% (225/253 bp), 88.53% (224/253 bp), and 88.53% (224/253 bp) sequence similarity, respectively. Sequences of the 16S rRNA and *gltA* genes from all 3 ticks were identical.

**Figure 7:**
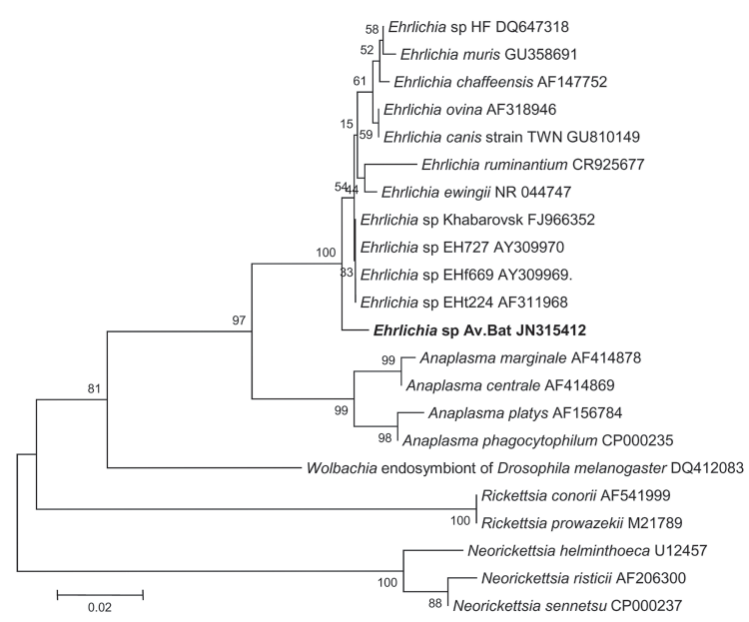
Phylogenetic trees drawn from an alignment of the 348-bp 16S rRNA gene specific to *Ehrlichia* spp. by using the minimum evolution method. Bootstrap values are indicated at the nodes. Scale bar indicate the degree of divergence represented by a given length of branch. **Boldface** indicates the taxonomic position of a new *Rickettsia* sp.

**Figure 8:**
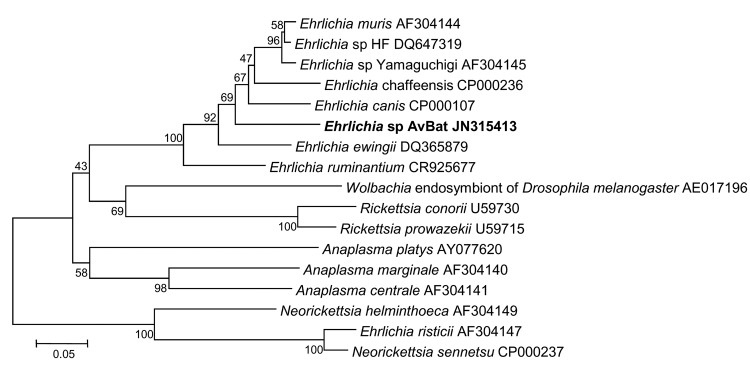
Phylogenetic trees drawn from an alignment of the 257-bp *gltA* gene specific to *Ehrlichia* spp. by using the minimum evolution method. Bootstrap values are indicated at the nodes. Scale bar indicate the degree of divergence represented by a given length of branch. **Boldface** indicates the taxonomic position of a new *Ehrlichia* sp.

We deposited nucleotide sequences for the 16S rRNA and *gltA* genes of this *Ehrlichia* sp. in GenBank (accession nos. JN315412 and JN315413, respectively). In phylogenetic trees based on 257 bp of the *gltA* gene and 348 bp of the 16S rRNA gene, this sequence is situated in the genus *Ehrlichia* in the *E. canis* group, and it is distinct from other known *Ehrlichia* spp. (Figures 7, 8) (26).

### Detection of *Bartonella* spp. and *C. burnetii*

We tested 5 ticks by qPCR for the presence of *Bartonella* spp. and *C. burnetii*. Results were negative for these bacteria.

### Co-infections

Of the 5 *A. vespertilionis* ticks analyzed by PCR, 4 were positive for >1 pathogen. Three of the 4 borreliae-positive ticks (nos. 62494, 62497, and 62498) were also infected with *Rickettsia* sp. AvBat. Two of the 3 *Ehrlichia* sp. AvBat–positive ticks (nos. 62495 and 62497) were also infected with *Borrelia* sp. CPB1, and 1 of those (no. 62497) was also infected with *Rickettsia* sp. AvBat.

## Discussion

We showed that *A. vespertilionis* ticks collected from a bat-infested attic in southwestern France were infected with 3 bacteria: 1) *Rickettsia* sp. AvBat, a new species or subspecies of the SFG rickettsiae; 2) a novel *Ehrlichia* sp. AvBat of the *E. canis* group of the genus *Ehrlichia*; and 3) *Borrelia* sp. from the relapsing fever group. 

In 1956, Hoogstraal (2) reported that several *A. vespertilionis* ticks from Egypt were examined for rickettsiae, and all were negative. Through sequence analysis of the full-length *gltA* gene, we showed that the *Rickettsia* sp. detected in *A. vespertilionis* ticks in France can be classified within the SFG rickettsiae (24).

The association of *Rickettsia spp*. with soft ticks is poorly understood. First, intracellular rickettsia-like symbionts were described in laboratory-maintained *Argas* (*Persicargas*) *arboreus* ticks, but the organisms have not been shown to infect vertebrates or cause disease (27). *Ornithodoros papillipes* ticks that were sucking blood from guinea pigs infected with *Rickettsia sibirica,* the agent of North Asian tick typhus, were found to be infected with the bacterium (28). *O. papillipes* ticks can transmit bacteria vertically but cannot transmit it to vertebrate hosts (28). In addition, in a laboratory experiment, *O. parkeri* and *O. rostratus* ticks were infected with *R. rickettsii*, the agent of Rocky Mountain spotted fever, and transmitted the bacterium to a laboratory host (29). In 1974, Rehácek et al. (30) found that *Argas persicus* ticks collected in Armenia were massively infected with SFG group rickettsiae that were probably identical with *R. slovaca*, an emerging pathogen (17). Rickettsiae-infected *O. moubata* ticks (well-known vectors of *Borrelia duttonii,* an agent of tick-borne relapsing fever) were collected from human dwellings in central Tanzania and analyzed by PCR; phylogenetic analysis of the rickettsial species showed a unique cluster among the SFG rickettsiae (31). At the same time, *R. felis,* the agent of the so-called flea-borne spotted fever (32), was detected in 1 of 64 *C. capensis* ticks collected from a brown pelican rookery in South Carolina, USA (33). A rickettsial endosymbiont, later named *R. hoogstraalii* sp. nov., was detected by use of PCR and isolated from *C. capensis* ticks (34,35) collected in the United States and Japan (33,36) and, later, from *Carios kelleyi* bat ticks collected from residential buildings in Jackson County, Iowa, USA (37). Transstadial and transovarial transmission of this rickettsia have also been demonstrated (33). Our results support further investigation of the association of soft ticks and rickettsiae.

The sequences for the *Borrelia* sp. detected in this study share 100% similarity with sequences for species detected in the liver of a dead bat in the United Kingdom in 2008 (25). Phylogenetic analysis showed that the *Borrelia* sp. detected in the bat is close to, but distinct from, borreliae in a cluster containing *B. recurrentis, B. duttonii,* and *B. crocidurae* (25), which all cause relapsing fever in Africa (13). In the study in the United Kingdom, an *A. vespertilionis* larval tick (not tested for borreliae by PCR) was found attached to the infected bat, and the study authors suggested that the tick may have been the source of *Borrelia* infection (25). Usually, only the larvae of *A. vespertilionis* ticks are found on bats because they feed on their hosts from the time they are a few days old up to 2 weeks of age. Nymphs and adults become replete within 1 hour; thus, the probability is small for finding ticks in these growth stages on bats (2). In addition, adults tick may remain attached for as long as 5 hours after completed engorgement. The results of our study confirm the association between *A. vespertilionis* ticks and this new *Borrelia* sp.

The presence of ehrlichiae was detected in 3 *A. vespertilionis* ticks in our study, but we did not culture the samples. The association between *Ehrlichia/Anaplasma* spp. and soft ticks is unknown. In 1990, Ewing et al. (38) reported that an *Otobius megnini* tick, which detached from the ear of a child who had serologic evidence of ehrlichiosis, was negative for ehrlichia by PCR. The authors of that study attempted to use laboratory-reared *O. megnini* ticks to transmit *E. canis,* the causative agent of canine ehrlichiosis; neither transstadial nor transovarial transmission occurred.

In our study, no residents of the bat-infested attic were bitten by ticks, although 2 persons were sleeping in the room the night before the ticks were collected. Adult and nymphal *A. vespertilionis* ticks occasionally bite humans, and they can be highly aggressive toward humans (39). Ticks of this species have been removed from persons in Iraq, the former Soviet Union, Japan, and Africa (2,3). In Huesca Province, Spain, 2 adult *A. vespertilionis* ticks were found feeding on the arm of a man inhabiting a country house with many bats living in the attic (40). In 1994, Jaenson et al. (3) reported that 2 persons living near Stockholm were bitten by ticks in their bedroom; bats had been breeding in a loft above the room during May–June 1993. Severe skin reactions, fever, ulceration, erythema, and edema developed in the bitten persons, and the ulcers did not begin to cicatrise until 10 days after penicillin treatment had been initiated. Thus, medical doctors should consider bacterial infections in sick person who may have been bitten by bat ticks.

The findings from our study have repercussions for public health in many parts of Europe, Asia, and Africa because *A. vespertilionis* ticks have a wide geographic range and may bite humans (2). Almost any bat, whether it lives in large colonies or in small groups, may be parasitized by *A. vespertilionis* ticks (2). We do not know whether the novel *Rickettsia* sp. AvBat and *Ehrlichia* sp*.* AvBat described in our work are pathogenic for vertebrate hosts; however, the *Borrelia* sp. detected in these ticks was shown to be a pathogen for bat hosts (25). In addition, the *Rickettsia* sp. AvBat that we cultivated in a cell line should be analyzed genomically to further define the taxonomic position of this *Rickettsia* sp. Future studies are needed to 1) assess the role of vector and/or reservoir for each of these pathogens in *A. vespertilionis*, including a more precise analysis of transstadial and transovarial transmission in ticks; 2) confirm tick transmission of the bacteria in animal models; and 3) detect tick transmission of these bacteria in humans.

## References

[1] Parola P, Raoult D. Ticks and tickborne bacterial diseases in humans: an emerging infectious threat. Clin Infect Dis. 2001;32:897–928. 10.1086/319347 11247714

[2] Hoogstraal H. African Ixodoidea: I. Ticks of the Sudan (with special reference to Equatoria Province and with preliminary reviews of the genera *Boophilus, Margaropus*, and *Hyalomma*). Washington (DC): US Navy; 1956. p. 1101.

[3] Jaenson TG, Talleklint L, Lundqvist L, Olsen B, Chirico J, Mejlon H. Geographical distribution, host associations, and vector roles of ticks (Acari: Ixodidae, Argasidae) in Sweden. J Med Entomol. 1994;31:240–56 . 8189415 10.1093/jmedent/31.2.240 PMC7107449

[4] Hoogstraal H. Notes on Egyptian ticks (Ixodoidea). I. The genus Argas (Argasidae) in the Cairo area. Proceedings of the Egyptian Academy of Sciences. 1952;7:114–27.

[5] Hoogstraal H. Bat ticks of the genus *Argas* (Ixodoidea, Argasidae). I. The subgenus *Chiropterargas.* Fieldiana. Zoology. 1955;37:579–600.

[6] Zhmaeva ZM, Pchelkina AA, Belashova VS. Spontaneous infection of *Argas vespertilionis* with *Rickettsia burnetii* in the south of Kazakhstan [in Russian]. Med Parazitol (Mosk). 1966;35:595–6 . 4238684

[7] Lvov DK, Karas FR, Timofeev EM, Tsyrkin YM, Vargina SG, Veselovskaya OV, “Issyk-Kul” virus, a new arbovirus isolated from bats and *Argas* (*Carios*) *vespertilionis* (Latr., 1802) in the Kirghiz S.S.R. Brief report. Arch Gesamte Virusforsch. 1973;42:207–9. 10.1007/BF01270841 4747048

[8] L’vov DK, Kostiukova MA, Pak TP, Gromashevskii VL. Isolation of an arbovirus antigenically related to Issyk-Kul virus from the blood of a human patient [in Russian]. Vopr Virusol. 1980; (Jan–Feb):61–2 . 6774483

[9] Gardner RA, Molyneux DH. *Babesia vesperuginis*: natural and experimental infections in British bats (Microchiroptera). Parasitology. 1987;95:461–9. 10.1017/S0031182000057887 3696774

[10] Hubbard MJ, Baker AS, Cann KJ. Distribution of *Borrelia burgdorferi* s.l. spirochaete DNA in British ticks (Argasidae and Ixodidae) since the 19th century, assessed by PCR. Med Vet Entomol. 1998;12:89–97. 10.1046/j.1365-2915.1998.00088.x 9513944

[11] Matsumoto K, Ogawa M, Brouqui P, Raoult D, Parola P. Transmission of *Rickettsia massiliae* in the tick, *Rhipicephalus turanicus.* Med Vet Entomol. 2005;19:263–70. 10.1111/j.1365-2915.2005.00569.x 16134974

[12] Socolovschi C, Mediannikov O, Sokhna C, Tall A, Diatta G, Bassene H, *Rickettsia felis*–associated uneruptive fever, Senegal. Emerg Infect Dis. 2010;16:1140–2. 10.3201/eid1607.100070 20587190 PMC3321914

[13] Parola P, Diatta G, Socolovschi C, Mediannikov O, Tall A, Bassene H, Tick-borne relapsing fever borreliosis, rural Senegal. Emerg Infect Dis. 2011;17:883–5. 10.3201/eid1705.100573 21529402 PMC3321757

[14] Mediannikov O, Fenollar F, Socolovschi C, Diatta G, Sokhna C, Bassene H, *Coxiella burnetii* in humans and ticks in rural Senegal. PLoS Negl Trop Dis. 2010;4:e654. 10.1371/journal.pntd.0000654 20386603 PMC2850317

[15] Raoult D, Roblot F, Rolain JM, Besnier JM, Loulergue J, Bastides F, First isolation of *Bartonella alsatica* from a valve of a patient with endocarditis. J Clin Microbiol. 2006;44:278–9. 10.1128/JCM.44.1.278-279.2006 16390990 PMC1351971

[16] Parola P, Roux V, Camicas JL, Baradji I, Brouqui P, Raoult D. Detection of ehrlichiae in African ticks by polymerase chain reaction. Trans R Soc Trop Med Hyg. 2000;94:707–8. 10.1016/S0035-9203(00)90243-8 11198664

[17] Sarih M, Socolovschi C, Boudebouch N, Hassar M, Parola P, Raoult D. Spotted fever group rickettsiae in ticks, Morocco. Emerg Infect Dis. 2008;14:1067–73. 10.3201/eid1407.070096 18598627 PMC2600325

[18] Assous MV, Wilamowski A, Bercovier H, Marva E. Molecular characterization of tickborne relapsing fever *Borrelia*, Israel. Emerg Infect Dis. 2006;12:1740–3. 10.3201/eid1211.060715 17283626 PMC3372360

[19] Parola P, Inokuma H, Camicas JL, Brouqui P, Raoult D. Detection and identification of spotted fever group rickettsiae and ehrlichiae in African ticks. Emerg Infect Dis. 2001;7:1014–7. 10.3201/eid0706.010616 11747731 PMC2631901

[20] Mediannikov OY, Sidelnikov Y, Ivanov L, Mokretsova E, Fournier PE, Tarasevich I, Acute tick-borne rickettsiosis caused by *Rickettsia heilongjiangensis* in Russian Far East. Emerg Infect Dis. 2004;10:810–7. 10.3201/eid1005.030437 15200813 PMC3323216

[21] Vestris G, Rolain JM, Fournier PE, Birg ML, Enea M, Patrice JY, Seven years’ experience of isolation of *Rickettsia* spp. from clinical specimens using the shell vial cell culture assay. Ann N Y Acad Sci. 2003;990:371–4. 10.1111/j.1749-6632.2003.tb07394.x 12860657

[22] Raoult D, Ndihokubwayo JB, Tissot-Dupont H, Roux V, Faugere B, Abegbinni R, Outbreak of epidemic typhus associated with trench fever in Burundi. Lancet. 1998;352:353–8. 10.1016/S0140-6736(97)12433-3 9717922

[23] Houhamdi L, Raoult D. Excretion of living *Borrelia recurrentis* in feces of infected human body lice. J Infect Dis. 2005;191:1898–906. 10.1086/429920 15871124

[24] Fournier PE, Dumler JS, Greub G, Zhang J, Wu Y, Raoult D. Gene sequence-based criteria for identification of new rickettsia isolates and description of *Rickettsia heilongjiangensis* sp. nov. J Clin Microbiol. 2003;41:5456–65. 10.1128/JCM.41.12.5456-5465.2003 14662925 PMC308961

[25] Evans NJ, Bown K, Timofte D, Simpson VR, Birtles RJ. Fatal borreliosis in bat caused by relapsing fever spirochete, United Kingdom. Emerg Infect Dis. 2009;15:1331–3. 10.3201/eid1508.090475 19751613 PMC2815988

[26] Dumler JS, Barbet AF, Bekker CPJ, Dasch GA, Palmer GH, Ray SC, Reorganization of genera in the families Rickettsiaceae and Anaplasmataceae in the order Rickettsiales: unification of some species of *Ehrlichia* with *Anaplasma, Cowdria* with *Ehrlichia* and *Ehrlichia* with *Neorickettsia*, descriptions of six new species combinations and designation of *Ehrlichia equi* and ‘HGE agent’ as subjective synonyms of *Ehrlichia phagocytophila.* Int J Syst Evol Microbiol. 2001;51:2145–65. 10.1099/00207713-51-6-2145 11760958

[27] El Shoura SM. Ultrastructure and distribution of intracellular rickettsia-like microorganisms in various organs of the laboratory-reared adult tick *Argas (Persicargas) arboreus* (Ixodoidea: Argasidae). Exp Appl Acarol. 1990;9:137–43. 10.1007/BF01198992 2226072

[28] Podboronov VM, Pchelkina AA. Characteristics of the transphase and transovarial transmission of *Rickettsia sibirica* by ixodid and argasid ticks [in Russian]. Med Parazitol (Mosk). 1989;4:14–8 . 2811744

[29] Hoogstraal H. Ticks in relation to human diseases caused by *Rickettsia* species. Annu Rev Entomol. 1967;12:377–420. 10.1146/annurev.en.12.010167.002113 5340723

[30] Rehácek J, Urvölgyi J, Kovácová E. Massive occurrence of rickettsiae of the spotted fever group in fowl tampan, *Argas persicus*, in the Armenian S.S.R. Acta Virol. 1977;21:431–8 . 22239

[31] Cutler SJ, Browning P, Scott JC. *Ornithodoros moubata*, a soft tick vector for *Rickettsia* in east Africa? Ann N Y Acad Sci. 2006;1078:373–7. 10.1196/annals.1374.074 17114744

[32] Parola P. *Rickettsia felis*: from a rare disease in the USA to a common cause of fever in sub-Saharan Africa. Clin Microbiol Infect. 2011;17:996–1000. 10.1111/j.1469-0691.2011.03516.x 21722253

[33] Reeves WK, Loftis AD, Sanders F, Spinks MD, Wills W, Denison AM, *Borrelia, Coxiella,* and *Rickettsia* in *Carios capensis* (Acari: Argasidae) from a brown pelican (*Pelecanus occidentalis*) rookery in South Carolina, USA. Exp Appl Acarol. 2006;39:321–9. 10.1007/s10493-006-9012-7 16821092

[34] Mattila JT, Burkhardt NY, Hutcheson HJ, Munderloh UG, Kurtti TJ. Isolation of cell lines and a rickettsial endosymbiont from the soft tick *Carios capensis* (Acari: Argasidae: Ornithodorinae). J Med Entomol. 2007;44:1091–101. 10.1603/0022-2585(2007)44[1091:IOCLAA]2.0.CO;2 18047211

[35] Duh D, Punda-Polic V, Avsic-Zupanc T, Bouyer D, Walker DH, Popov VL, *Rickettsia hoogstraalii* sp. nov., isolated from hard- and soft-bodied ticks. Int J Syst Evol Microbiol. 2010;60:977–84. 10.1099/ijs.0.011049-0 19666817

[36] Kawabata H, Ando S, Kishimoto T, Kurane I, Takano A, Nogami S, First detection of *Rickettsia* in soft-bodied ticks associated with seabirds, Japan. Microbiol Immunol. 2006;50:403–6 . 16714848 10.1111/j.1348-0421.2006.tb03807.x

[37] Loftis AD, Gill JS, Schriefer ME, Levin ML, Eremeeva ME, Gilchrist MJR, Detection of *Rickettsia, Borrelia*, and *Bartonella* in *Carios kelleyi* (Acari: Argasidae). J Med Entomol. 2005;42:473–80. 10.1603/0022-2585(2005)042[0473:DORBAB]2.0.CO;2 15962801

[38] Ewing SA, Harkess JR, Kocan KM, Barker RW, Fox JC, Tyler RD, Failure to transmit *Ehrlichia canis* (Rickettsiales: Ehrlichieae) with *Otobius megnini* (Acari: Argasidae). J Med Entomol. 1990;27:803–6 . 2231616 10.1093/jmedent/27.5.803

[39] Hoogstraal H. Argasid and nuttalliellid tick as parasites and vectors. Adv Parasitol. 1985;24:135–238. 10.1016/S0065-308X(08)60563-1 3904345

[40] Estrada-Peña A, Jongejan F. Ticks feeding on humans: a review of records on human-biting Ixodoidea with special reference to pathogen transmission. Exp Appl Acarol. 1999;23:685–715. 10.1023/A:1006241108739 10581710

